# Facile preparation of acid-resistant magnetite particles for removal of Sb(Ⅲ) from strong acidic solution

**DOI:** 10.1080/14686996.2016.1145530

**Published:** 2016-03-16

**Authors:** Dong Wang, Kaiwen Guan, Zhiping Bai, Fuqiang Liu

**Affiliations:** ^a^ State Key Laboratory of Coordination Chemistry, School of Chemistry and Chemical Engineering, Nanjing University, Nanjing210023, P.R. China; ^b^ State Key Laboratory of Pollution Control and Resource Reuse, School of the Environment, Nanjing University, Nanjing210046, P.R. China

**Keywords:** Magnetite nanoparticles, acid-resistant, sol-gel, adsorption

## Abstract

A new facile coating strategy based on the hydrophobicity of methyl groups was developed to prevent nano-sized magnetite particles from strong acid corrosion. In this method, three steps of hydrolysis led to three layers of protection shell coating Fe_3_O_4_ nanoparticles. Filled with hydrophobic methyl groups, the middle layer mainly prevented the magnetic core from strong acid corrosion. These magnetite particles managed to resist 1 M HCl solution and 2.5 M H_2_SO_4_ solution. The acid resistant ability was higher than those reported previously. After further modification with amino-methylene-phosphonic groups, these magnetite particles successfully adsorbed Sb(III) in strong acid solution. This new strategy can also be applied to protect other materials from strong acid corrosion.

## Graphical abstract

**Figure:**
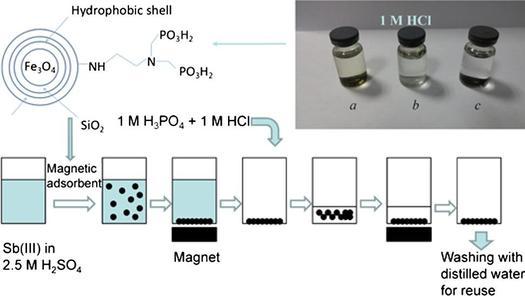

## 1. Introduction

In the past few years, magnetic solid phase extraction (MSPE) with magnetic particles as adsorbents has been applicable to metal removal and trace-metal analysis [1–5]. Compared with conventional solid phase extraction, sample pretreatment is substantially simplified by employing magnetic adsorbents because there is no need for packing a column with sorbents in batch operation. Instead, phase separation can be quickly and easily accomplished by an external magnetic field. Among the magnetic particles used in MSPE, micro- and nano-sized magnetite (Fe_3_O_4_) particles, which can be dissolved in strong acid solutions (pH < 1), are most popular [6].

Magnetite particles have been most commonly coated by silica gel, considering mechanical and chemical stabilities as well as thermal resistance under various conditions [7]. In addition, this method enjoys easy surface modification and control of interparticle interactions [8,9]. However, silica gel-coated Fe_3_O_4_ particles still cannot be applied in strong acid conditions because Fe_3_O_4_ cores are vulnerable to corrosion. Li et al. [10] and Liu et al. [11] prevented Fe_3_O_4_ cores from acid corrosion by employing dense liquid coating which required the reaction temperature to be maintained at 90°C. It is not convenient for coating Fe_3_O_4_ particles though, as nano-sized Fe_3_O_4_ particles are prone to conversion into Fe_2_O_3_ at high temperature when exposed to the atmosphere [12–14]. Besides, although these silica-coated materials can stand 0.1 M HCl, strong acid conditions are still troublesome. Therefore, the challenge remains to develop a suitable coating strategy that protects magnetite particles from strong acid corrosion by silica gel.

In recent years, silica-coated Fe_3_O_4_ particles have been modified with various functional groups to remove different metal ions from wastes and water, such as Pd(II) [15], Cr(III) [16–18], Pb(II) [19,20], Ag(I) [21] and Cd(II) [22]. Since the existing coating technologies using silica gel fail to resist acids, corresponding magnetite particles have not been successfully used to adsorb dissociated metal ions under strong acid conditions.

On the other hand, some kinds of metal ion need to be removed from strong acid solutions or waste liquids simultaneously in industrial practice. For instance, removal of antimony plays an essential role in treating electrolytes of copper electro-refining [23,24]. The electrolyte is a solution of copper sulfate and sulfuric acid whose nominal composition is 40–45 g l^–1^ copper and 180–200 g l^–1^ sulfuric acid. In the past, numerous materials have been successfully developed to separate antimony species from water, such as hydroxyapatite [25], graphene [26], diatomite [27], goethite [28], bentonite [29], Fe-Mn binary oxide [30], raw and modified multi-walled carbon nanotubes [31] and hematite-coated magnetic nanoparticles [32]. However, these materials may not be suitable for removing Sb in copper refining due to the strong acid conditions of electrolyte and the demand for recycling. Meanwhile, silica gel-coated magnetic nanoparticles have not been applied in strong acid conditions either. In the industry, some ion-exchange resins possessing an amino-phosphonic acid functional group, including Eporus MX-2 (Miyoshi Oil and Fat Company, Tokyo, Japan), Unitika UR-3300 (Unitika Ltd, Osaka, Japan) and Duolite C-467 (Rohm and Haas Company, Philadelphia, USA), have been used to remove Sb and then recycled by being eluted with 6 M HCl [24]. Therefore, developing a new strategy that coats magnetite (Fe_3_O_4_) particles by silica gel is bound to expand the application of MSPE by preventing strong acid corrosion.

In this study, a new facile coating method based on the traditional sol-gel process was developed and operated at only 30°C, depending significantly on the hydrophobicity of methyl groups. After coating, this material managed to stand the corrosion by 1 M HCl solution for 24 h. To prove the practicability of the acid-resistant magnetite nanoparticles, amino-methylene-phosphonic groups were fixed onto their surfaces to adsorb Sb(III) in acid condition. These groups have been applied to remove Sb(III) from copper electro-refining electrolytes [33]. As we expected, this novel magnetic adsorbent successfully removed Sb(III) from 2.5 M H_2_SO_4_ solution, leaving no ferric ions in the supernatant and keeping the cores stable.

## 2. Experimental section

### 2.1. Materials

FeSO_4_·7H_2_O, Fe_2_(SO_4_)_3_, NaOH, HCl, H_2_SO_4_, formaldehyde and tetraethyl orthosilicate (TEOS) were all guaranteed reagents from Nanjing Chemical Reagent Co., Ltd. (Nanjing, China) Dimethyldiethoxysilane (DMDES), (3-aminopropyl China)triethoxysilane (APTES), 2-choroethylamine hydrochloride and Sb_2_O_3_ were purchased from Aladdin Industrial Corporation (Ontario, Canada) Sb_2_O_3_ was dissolved in concentrated sulfuric acid to prepare Sb(III) solution. All the chemicals were used directly without any further purification. Nano-sized ultrafine magnetic Fe_3_O_4_ powders were prepared by the coprecipitation method [14]. Referring to a previous work [34], N-(2-chloroethyl)iminobis(methylene)diphosphonic acid was synthesized from 2-choroethylamine hydrochloride with a yield of 87%.

### 2.2. Preparation of ultrafine magnetic Fe_3_O_4_ powders

FeSO_4_·7H_2_O (5.6 g) and Fe_2_(SO_4_)_3_ (8 g) were dissolved into 100 ml of distilled water and stirred until the mixture became an orange colloid. Then 15 g NaOH was added into 200 ml of distilled water with strong stirring. When the NaOH solution was cooled down to room temperature, the orange colloid was dropwise added over one hour with continuous stirring. After the reaction, the black mixture was separated by being centrifuged at 5000 rpm. Then, the black precipitate was washed three times with distilled water (100 ml). Finally, the ultrafine Fe_3_O_4_ powders were dispersed in 150 ml of pure ethanol for later use.

### 2.3. Coating Fe_3_O_4_ powders by sol-gel process

In a typical experiment, a mixture of Fe_3_O_4_ powders dispersed in 150 ml of ethanol was sonicated for 30 min first. Then 25 ml of distilled water and 1 ml of aqueous ammonia were added into the mixture successively with strong stirring to adjust pH. Meanwhile, before the reagents were added, the temperature of the water bath was adjusted to 30°C and maintained until the experiment ended. Fifteen minutes later, 10 ml of TEOS was injected into the mixture, followed by reaction for one hour. Before further treatment, a small amount of particles were separated from the reaction system as sample *a*. After 1 ml of aqueous ammonia was injected again, 10 ml of TEOS and 6 ml of DMDES were added into the flask, and the reaction was maintained for 4 h. Then, by employing an external magnetic field, the black powders were easily separated from the mixture, and washed three times with ethanol (50 ml). Next, these cleaned magnetic powders were dispersed into 150 ml of ethanol and 25 ml of pure water again. After 1 ml of aqueous ammonia and 6 ml of DMDES were injected in sequence, the reaction was maintained under strong stirring for 24 h at 30°C. Finally, the magnetic Fe_3_O_4_ particles were successfully coated with a layer of hydrophobic silica. Magnetic field allowed easy phase separation and washing of these particles. After this coating process, the samples with a structure of Fe_3_O_4_@SiO_2_@SiO-Me were marked as sample *b*.

### 2.4. Modification of amino group

Previous magnetic Fe_3_O_4_@SiO_2_@SiO-Me particles were dispersed into 150 ml of ethanol and 25 ml of pure water again, into which was added 1 ml of aqueous ammonia under strong stirring at 30°C. Then 10 ml of TEOS was added into the flask. Twenty minutes later, 4 ml of APTES was poured into the flask and the reaction was maintained for 4 h. Last, the products marked as sample *c* with a structure of Fe_3_O_4_@SiO_2_@SiO-Me@SiO-NH_2_ were separated by external magnetic field and washed by ethanol (50 ml) twice and distilled water (100 ml) three times in order.

### 2.5. Modification of amino-methylene-phosphonic group

N-(2-chloroethyl)iminobis(methylene)diphosphonic acid (1 g) was dissolved in 50 ml of distilled water at below 10°C. Then, the acid solution was neutralized by adding 1 M NaOH solution dropwise until pH = 7. After this solution was poured into a flask, 5 g magnetic Fe_3_O_4_@SiO_2_@SiO-Me@SiO-NH_2_ was dispersed into the solution and heated to 70°C with stirring. One hour later, the flask was evacuated, with the temperature maintained at 70°C. After vacuum drying, the products were washed by 20 ml of 1 M HCl twice and distilled water (50 ml) three times in sequence. Finally, the products were dried in a vacuum drying oven at 50°C.

### 2.6. Characterization of coated magnetite particles

Morphology and size of coated magnetite particles were determined by transmission electron microscopy (TEM, JEM-200CX, JEOL Ltd, Tokyo, Japan). Zeta potentials of the prepared sorption material were measured by Zetasizer Nano-Z (Malvern Instruments, Worcestershire, UK). Element compositions of the shell were investigated by elemental analyzer (Heraeus CHN-O-Rapid Heraeus Group, Hanau, Germany). The X-ray diffraction (XRD) patterns were collected on a D8 ADVANCE diffractometer with Cu Kα radiation (Bruker Corporation, Billerica, USA). An ASAP2020 physisorption analyzer (Micromeritics, Norcross, USA) was employed to measure the specific surface areas.

### 2.7. Leaching test

To examine the coverage and property of the coating, 0.05 g Fe_3_O_4_@SiO_2_@SiO-Me@SiO-NH_2_ particles were dispersed into 25 ml of 1 M HCl acid solution. Twenty-four hours later, the suspension was separated by external magnetic field. The supernatant was collected and analyzed for ion concentration by atomic absorption spectroscopy (Hitachi 180-80, Hitachi Ltd, Tokyo, Japan).

### 2.8. Batch adsorption experiments

In batch adsorption experiments, 0.05 g Fe_3_O_4_@SiO_2_@SiO-Me@SiO-NH_2_ particles modified with amino-methylene-phosphonic groups were dispersed into 25 ml of Sb(III) solution with 2.5 M H_2_SO_4_ in a 100 ml conical flask under ultrasonic wave for 2 min. The flasks were shaken at 100 rpm and 25°C in a constant-temperature shaking table for 6 h to reach equilibrium. Adsorption isotherm at 25°C was obtained by varying the initial Sb(III) concentration from 0.01 to 0.5 g l^–1^. After reaching the adsorption equilibrium, the mixture was separated by external magnetic field to collect supernatant. The Sb(III) concentration of supernatant was analyzed by atomic absorption spectroscopy.

### 2.9. Selective adsorption experiments

To investigate the adsorption performance in copper electro-refining electrolytes, a series of solutions containing 14.115 g l^–1^ Cu(II) and 2.5 M H_2_SO_4_ with different concentrations of Sb(III) were prepared. In a typical experiment, 0.05 g adsorbent was dispersed into 25 ml of simulated electrolytes in a conical flask. The flasks were shaken at 100 rpm and 25°C in a constant-temperature shaking table for 6 h to reach equilibrium. Then, after the suspension was separated by magnetic field, the supernatant was obtained to analyze the contents of Cu(II) and Sb(III) respectively.

## 3. Result and discussion

### 3.1. Characterization

**Figure 1.**
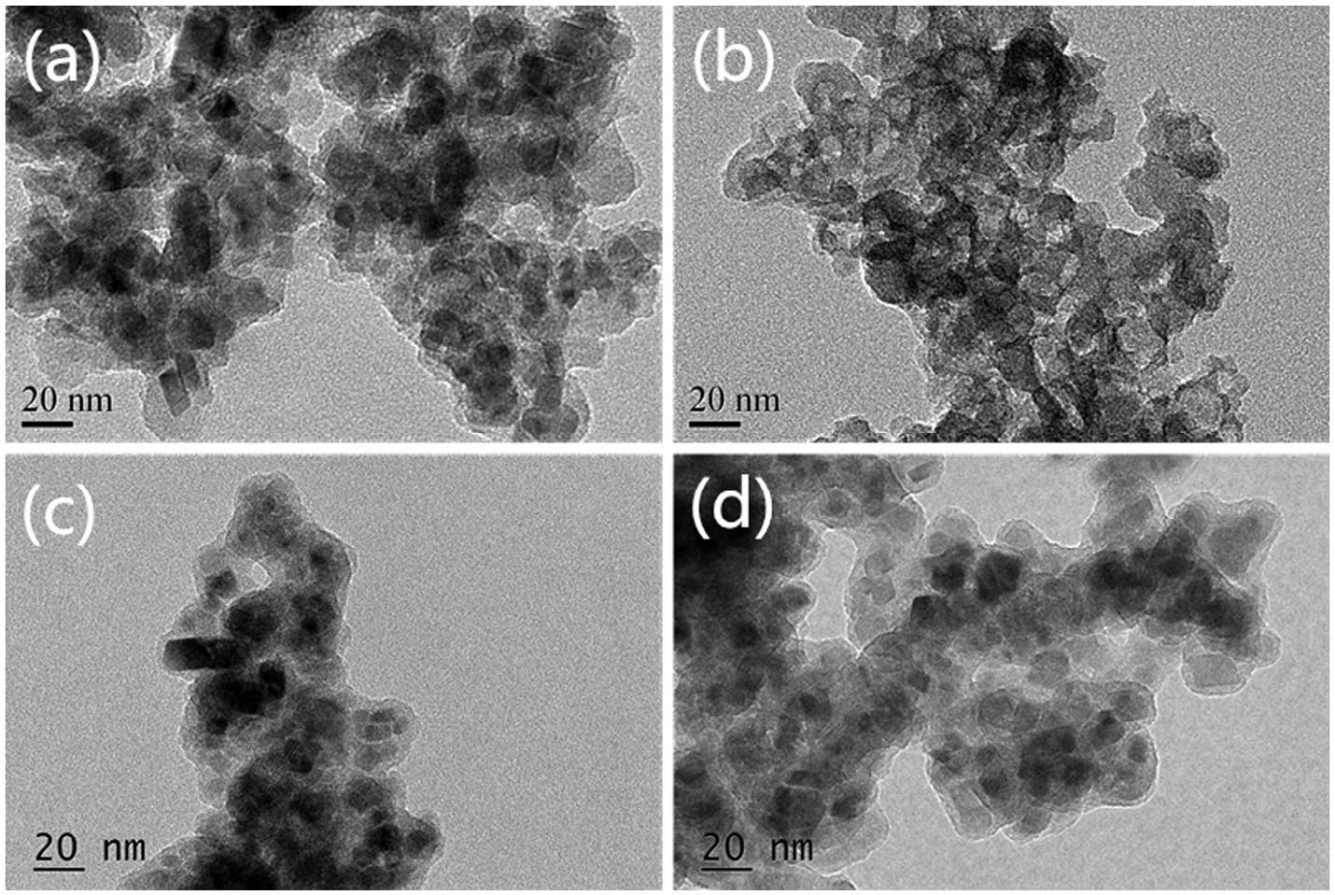
TEM images of (a) acid-resistant magnetic Fe_3_O_4_ nanoparticles; (b) shell of the sorbent; (c) sample *a*; (d) sample *b*.

To investigate the shell structure of Fe_3_O_4_@SiO_2_@SiO-Me@SiO-NH_2_, these particles were mixed with 6 M HCl at room temperature to remove the magnetic core, leaving white shell powders. The shells without cores were also determined by TEM and compared with normal samples. As shown in Figure 1(a), the magnetic cores with irregular morphologies are sized 10–20 nm. The morphologies of the shells without magnetic cores are exhibited in Figure 1(b). Obviously, the shell, with the core retained, was about 10 nm thick, which may partly be responsible for protecting the magnetic core from strong acid corrosion. In order to clarify the increase in particle size and to evidence the shell formation in different coating steps, samples *a* and *b* were also detected by TEM, as shown in Figure 1(c) and (d). The shell thickness of sample *b* (nearly 5 nm) resembled that of sample *a*, suggesting that coating of methyl groups did not significantly raise the particle size. SiO_2_ shell coating and amino group modification markedly increased the shell thickness and the particle size (Figure 1(a), (c) and (d)). Although the hydrophobic shell played a main role in resisting acids, it could hardly be distinguished by TEM. Therefore, whether the particles had been coated with the hydrophobic methyl groups remained uncertain, for which elemental analysis was performed. To investigate the structure of the coated magnetic Fe_3_O_4_ particles, samples *a*–*c* were collected successively from the reaction system after SiO_2_ shell coating, hydrophobic shell coating and amino group modification. The elemental analysis results of samples *a*–*c* are listed in Table 1.

**Table 1.** Elemental analysis (in wt%) of samples *a*-*e*.

Sample	C	H	N
*a*	0.31	0.5	0.08
*b*	1.42	0.8	0.07
*c*	6.89	2.2	2.13
*d*	1.51	0.9	0.08
*e*	1.89	1.2	0.09

**Figure 2.**
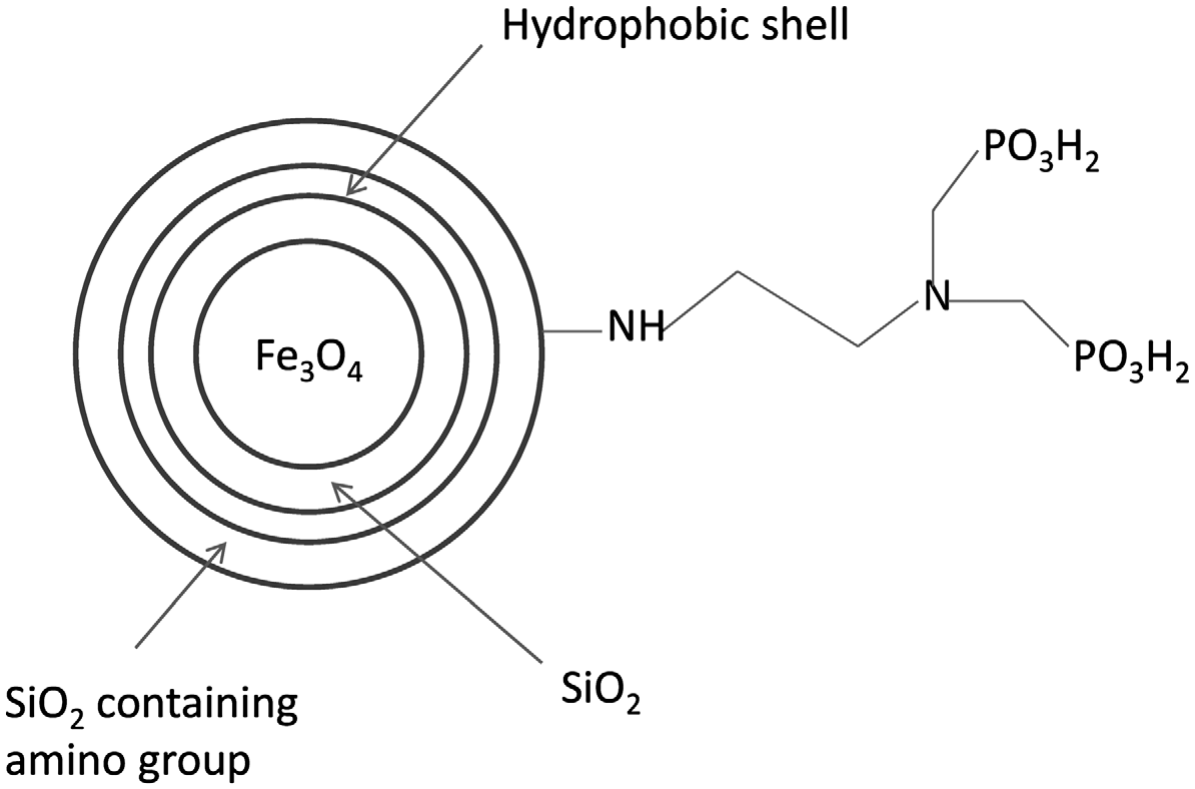
Schematic diagram of the structure of acid-resistant magnetic Fe_3_O_4_ nanoparticles.

In sample *a*, the total content of elements C and H that may originate from residual ethanol and water was below 1%. Significant increase in the mass percent of element C in sample *b* demonstrated that methyl groups had been successfully modified on the surface of magnetic particles after coating of hydrophobic shell by using DMDES. In other words, the particles can be coated with the hydrophobic shell containing methyl groups by our method. But the increase range was not enough to reflect the increase in shell thickness. The C and N contents of sample *c* both obviously exceeded those of samples *a* and *b*. This fact implies the shell was rapidly thickened during modification of amino group, agreeing with the TEM result. According to the above analysis, the acid-resistant magnetic Fe_3_O_4_ nanoparticles were structurally identified (Figure 2).

To evaluate the effects of experimental operation on hydrophobic shell coating with methyl groups, other two comparison experiments were designed. In one of them, during the coating of hydrophobic methyl groups, 10 ml of TEOS and 6 ml of DMDES were added into the reaction system, 30 min after which another 6 ml of DMDES was added, and the reaction was then maintained for 4 h. The samples coated by this method were marked as sample *d*. In the other experiment, the magnetite particles were coated through hydrolysis of 10 ml of TEOS and 6 ml of DMDES in the same way twice during the coating of hydrophobic protection shell, giving sample *e*. Like samples *a*–*c*, samples *d* and *e* were also detected by elemental analysis.

During the coating of methyl groups, DMDES needs to be hydrolyzed with TEOS at the same time, because DMDES alone can hardly fix methyl groups on the surface of magnetite particles. On the other hand, the elemental analysis results of samples *b* and *d* showed that another 6 ml of DMDES did not markedly increase the carbon content. In other words, methyl groups were barely fixed on the particle surface when only DMDES hydrolyzed. Meanwhile, the elemental analysis results of samples *e* and *b* indicated that hydrolysis of DMDES with TEOS twice brought more hydrophobic methyl groups than those in sample *b*. However, the second hydrolysis did not increase the carbon content as the first one did.

As shown in Figure 1(a), all the coated magnetite particles seem to aggregate. In order to detect the dispersity of the particles, the specific surface area was measured before and after coating by BET method. In theory, the specific surface area of pure ultrafine Fe_3_O_4_ powders without coating with a diameter of 10 nm reaches 116 m^2^ g^–1^, and the experimental value was 107 m^2^ g^–1^, suggesting that the magnetite powders used in our study had a high dispersity. Meanwhile, the magnetite particles after coating had a specific surface area of 60 m^2^ g^–1^. Given that the surface area was inversely proportional to the particle diameter, the dispersity was still acceptable.

These acid-resistant magnetite particles were herein applied in a strong acid condition with 2.5 M H_2_SO_4_, so the zeta-potential of amino-methylene-phosphonic group-modified particles was measured in the same solution. As suggested by the actual value (+52.7 mV), these particles were quite stable in this condition.

### 3.2. Leaching test

After sample *c* was immersed in 1 M HCl solution for 24 h, atomic absorption spectroscopic results showed that the supernatant contained no ferric ions, verifying that these magnetite nanoparticles coated with hydrophobic methyl groups managed to resist 1 M HCl solution, which was notably superior to the outcomes of previous studies. Hence, our coating method may render magnetic nano- or micro-sized metal oxide particles applicable to strong acid conditions.

**Figure 3.**
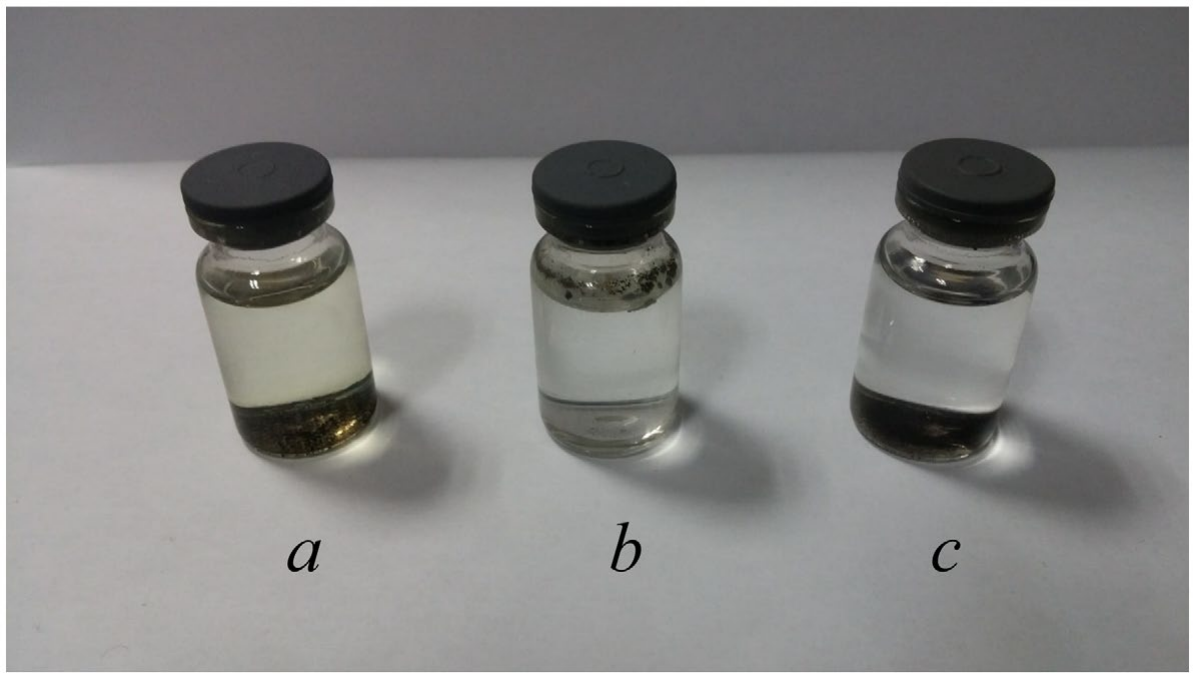
Samples *a*, *b* and *c* after being added in 1 M HCl solution for 24 h.

Samples *a*, *b* and *c* (0.01 g each) were respectively dispersed into 7 ml of 1 M HCl solution, 24 h after which color changes of the supernatants were observed (Figure 3). The supernatant of sample *a* turned yellow, indicating that ferric ions were leached into the solution. In other words, coating only one layer of SiO_2_ failed to prevent the cores from corrosion by strong acid. Nevertheless, the innermost layer of SiO_2_ was essential and indispensable for further coating. Furthermore, sample *b* kept floating above the acid solution, so the outside was full of hydrophobic methyl groups that hindered the particles from being dispersed into the acid solution. Since the supernatant of sample *c* was still colorless after 24 h, it had high acid resistance. In addition, the outmost layer, which was full of amino groups, benefited from further modification and generated a hydrophilic outside surface as well.

As mentioned above, copper electro-refining electrolyte usually contains 180-200 g l^–1^ sulfuric acid. Hence, we also used 2.5 M H_2_SO_4_ solution to test our acid-resistant magnetite particles. As expected, these magnetite particles stood the erosion of 2.5 M H_2_SO_4_ solution, because there were no ferric ions in the supernatant. For the sake of recycling, the coated magnetic Fe_3_O_4_ particles may have to resist the corrosion by stronger acid, such as 3 M HCl solution. The concentration of iron ions in the supernatant reached 1.7 mg l^–1^ one hour after these coated magnetite particles were immersed in 3 M HCl. Two hours later, such concentration rapidly increased to 15.6 mg l^–1^, meaning that 3 M HCl penetrated the shell to erode the magnetic core. Three hours later, the color of the supernatant, as observed by naked eyes, turned green. Although these coated magnetite particles could not resist 3 M HCl solution, the leaching concentration of iron ions at one hour was acceptable, i.e. the leached ferric ions were negligible during short-term contact.

**Figure 4.**
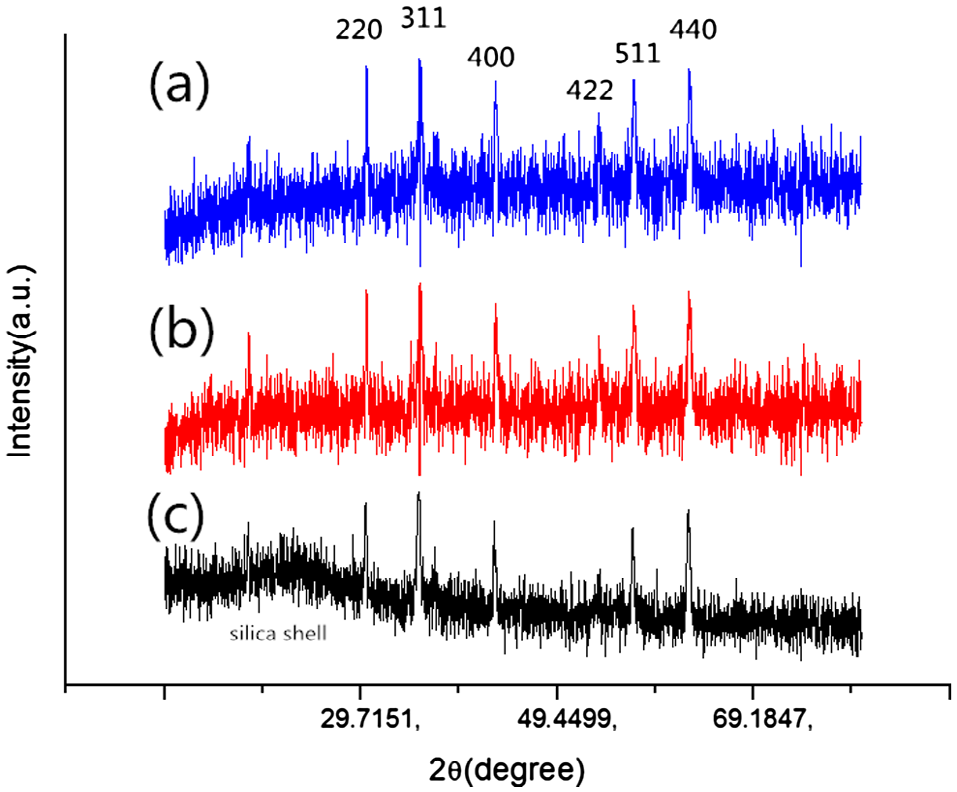
XRD patterns of (a) acid-resistant magnetic Fe_3_O_4_ nanoparticles; (b) acid-resistant magnetic Fe_3_O_4_ nanoparticles contacting with 1 M HCl for 24 h; (c) acid-resistant magnetic Fe_3_O_4_ nanoparticles contacting with 3 M HCl for 24 h.

To prove the stability of coated magnetic particles in strong acid conditions, a powder X-ray diffraction study was performed to investigate the changes of the adsorbent before and after contacting with acid solution. As shown in Figure 4, the XRD pattern of acid-resistant magnetic Fe_3_O_4_ nanoparticles shows characteristic (220), (311), (400), (422), (511) and (440) peaks. The XRD pattern is in good agreement with the data for cubic Fe_3_O_4_ as reported in the JCPDS card (No. 88-315, α = 8.375). Thus, this coating strategy did not undermine the property of the magnetic cores. On the other hand, the coated magnetite particles after contacting with 1 M HCl for 24 h have similar peaks to those before contact (Figure 4(a) and (b)), indicating that the magnetic Fe_3_O_4_ cores were well retained after contact, and that the hydrophobic shells successfully protected the cores from strong acid corrosion. In addition, the coated magnetite particles after contacting with 3 M for 24 h were also detected by XRD (Figure 4(c)). Obviously, the pattern of the sample contacting with 3 M HCl is different from others. In other words, contacting with 3 M HCl solution for 24 h dissolved part of the magnetite cores. In fact, the black coated magnetite powders turned gray after such contact.

### 3.3. Effect of contact time

**Figure 5.**
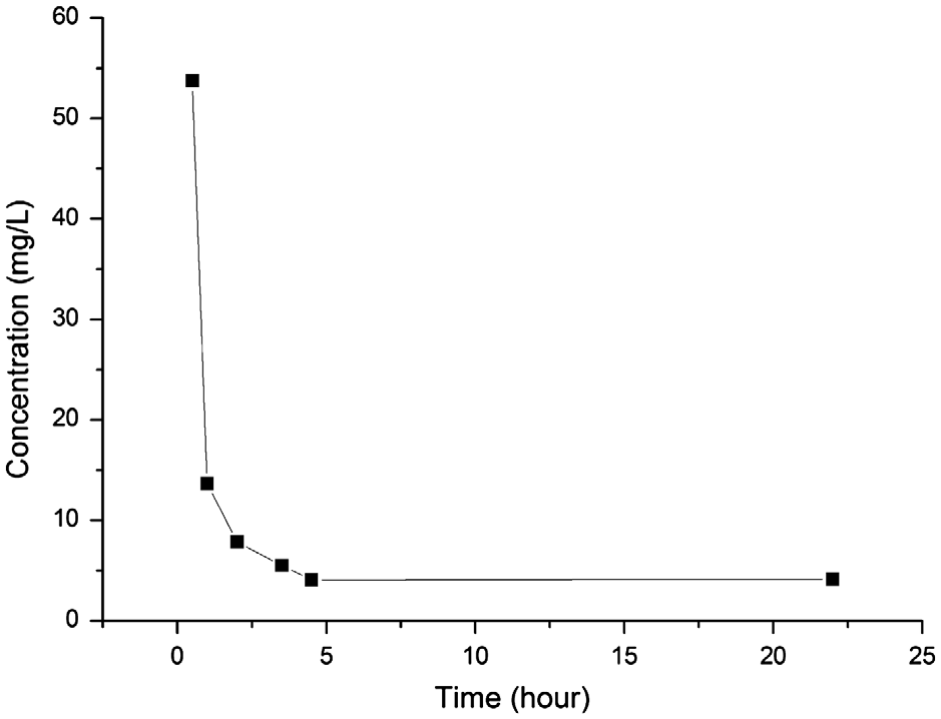
Removal of Sb(III) from 2.5 M H_2_SO_4_ solution. Initial concentration of Sb(III): 133 mg l^–1^; adsorbent dosage: 2 g l^–1^.

Fine powders are characterized by large specific surface areas and the resultant high adsorption speeds. In a previous work, however, the adsorbent with the same chelating group took 2 h to completely adsorb free metal ions in the acid solution. Thus, the effect of contact time was evaluated to clarify the adsorption equilibrium time of our magnetic Fe_3_O_4_@SiO_2_@SiO-Me@SiO-NH_2_ particles modified with amino-methylene-phosphonic groups. In a typical experiment, 0.15 g adsorbent was dispersed into 75 ml of Sb(Ⅲ) solution with 2.5 M H_2_SO_4_ in a 300 ml conical flask. The initial concentration of Sb(Ⅲ) was 133 mg l^–1^. The conical flask was placed in a constant-temperature shaking table with a speed of 100 rpm at 25°C. At different time intervals, 1 ml of supernatant was collected to analyze the residual Sb(Ⅲ) concentration (Figure 5).

In the beginning, the concentration of Sb(III) dropped quickly, implying the adsorption was fast initially. The adsorption of Sb(III) from 2.5 M sulfuric acid solution spent four hours to reach equilibrium. Given that the adsorption took 2 h of contact to remove most of antimony (Ⅲ), a specific surface area of 60 m^2^ g^–1^ did accelerate the adsorption of Sb(III) by using the nano-sized magnetite particles modified with amino-methylene-phosphonic groups.

### 3.4. Adsorption isotherm

**Figure 6.**
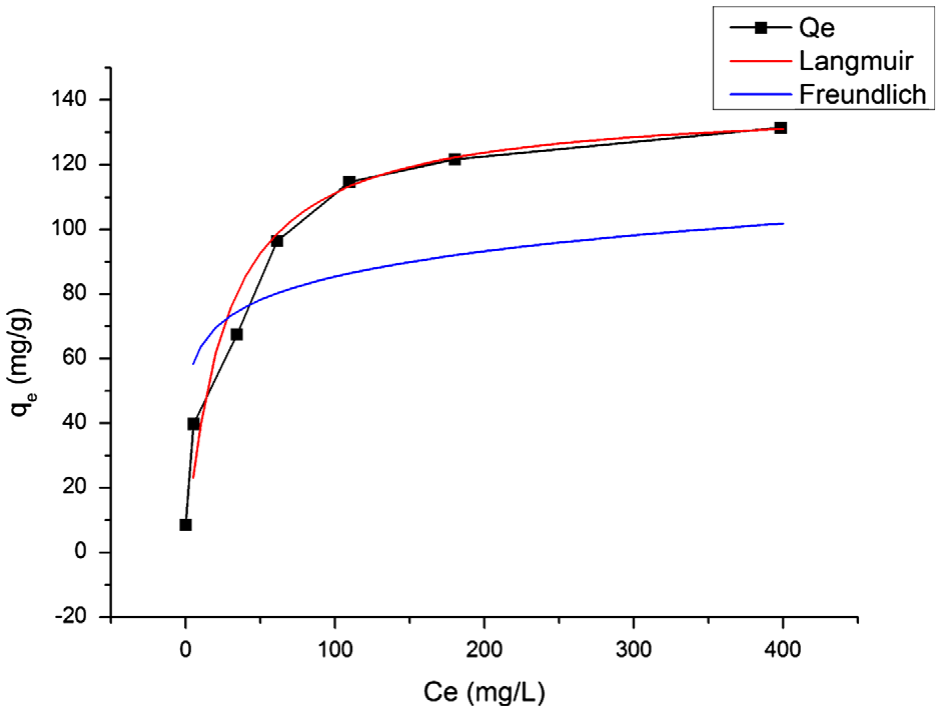
Sb(III) adsorption isotherms of acid-resistant magnetic Fe_3_O_4_ nanoparticles at 25°C in 2.5 M H_2_SO_4_ solution. Adsorbent dosage: 2 g l^–1^; contact time: 6 h.

Sb(III) adsorption isotherm of our acid-resistant magnetic nanoparticles in 2.5 M H_2_SO_4_ solution was obtained at 25°C by varying the initial concentration of Sb(III) from 0.01 g l^–1^ to 0.5 g l^–1^ (Figure 6). The adsorption isotherm was fitted with the Langmuir model and the Freundlich model as Equations (1) and (2), respectively.

(1) $$q_{e}=\frac{q_{m}K_{L}C_{e}}{1+K_{L}C_{e}}$$

(2) $$q_{e}=K_{F}C_{e}^{1/n}$$

where *C*_*e*_ is the equilibrium concentration of Sb(III) in mg l^–1^, and *q*_*e*_ is the amount of Sb(III) adsorbed at equilibrium in mg l^–1^. In Equation (1), *q*_*m*_ (mg g^–1^) indicates the adsorption capacity, whereas *K*_*L*_ (l mg^–1^) is the Langmuir affinity constant related to the energy of adsorption. In Equation (2), *K*_*F*_ is defined as the Freundlich adsorption affinity, while *n* is an index related to the heterogeneity of the adsorbent surface.

The Langmuir isotherm [35] assumes that sorption occurs on a structurally homogeneous adsorbent and all the adsorption sites are energetically identical. It is characterized by a decreasing slope as the concentration increases, since vacant adsorption sites decrease as the adsorbent is gradually covered. The Freundlich isotherm [36] is derived by assuming a heterogeneous surface with a non-uniform distribution of heat of adsorption over the surface and reversible adsorption. This model allows coexistence of several kinds of sorption sites on the solid and represents properly the sorption data at low and intermediate concentrations on heterogeneous surfaces. As shown in Figure 6, the Langmuir model can fit the experiment results more precisely than the Freundlich model does. Therefore, the adsorption process followed the Langmuir isotherm.

### 3.5. Selective adsorption of Sb(III) from strongly acid CuSO_4_ solution

**Figure 7.**
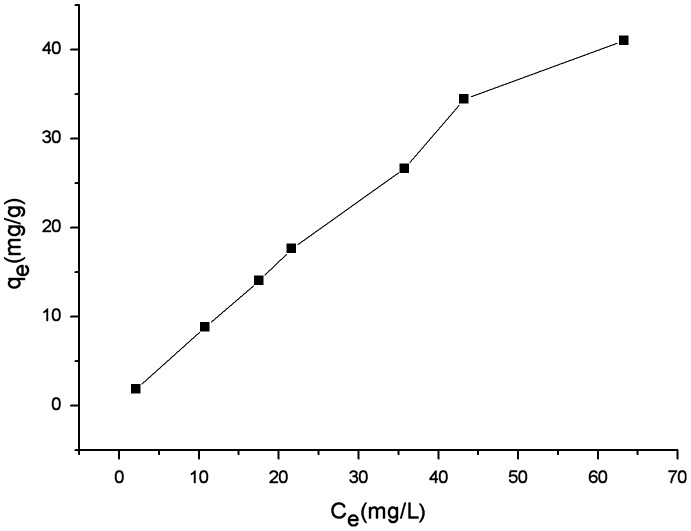
Selective removal of Sb(III) from cupric sulfate ([Cu(II)]: 14.115 g l^–1^) solution with 2.5 M H_2_SO_4_ at 25°C. Adsorbent dosage: 2 g l^–1^; contact time: 6 h.

The adsorptive capacity of Sb(III) with different initial concentrations in a CuSO_4_ solution ([Cu(II)]: 14.115 g l^–1^) with 2.5 M H_2_SO_4_ was further studied. Referring to the batch adsorption experiment, 0.05 g sorbents were dispersed into 25 ml of this solution. Two hours later, the concentrations of Sb (III) and Cu^2+^ in the supernatant were both detected. The concentrations of Sb (III) with different initial concentrations were all reduced after adsorption, while those of Cu^2+^ remained unchanged. As shown in Figure 7, the acid-resistant magnetic sorbent modified with amino-methylene-phosphonic chelating group can selectively remove Sb(III) from strongly acid CuSO_4_ solution. Hence, our sorbents are practically eligible for removal of Sb(III) from strongly acid copper electro-refining electrolytes.

### 3.6. Recovery of the adsorbent

Magnetic Fe_3_O_4_ nanoparticles have been utilized to remove metal ions from a water system, but they have seldom been reused because desorption of metal ions needs washing by using strong acids. For cost concerns, the acid-resistant magnetic Fe_3_O_4_ sorbent coated with hydrophobic shell was herein recycled and reused three times.

In a recycle, 0.1 g sorbents were dispersed into 25 ml of cupric sulfate ([Cu(II)]: 14.876 g l^–1^) solution with 175.84 ppm Sb(III) and 2.5 M H_2_SO_4_, and the mixture was shaken at 100 rpm and 25°C. Two hours later, the sorbents were separated from the mixture by external magnetic field, and the supernatant was collected to measure the concentration of Sb(III). Then the sorbents were washed by 10 ml of distilled water three times. Next, these sorbents were dispersed into 10 ml of 1 M HCl and 1 M H_3_PO_4_ eluent, and shaken for 30 min. After the eluate was collected, the elution was repeated using 10 ml of the same eluent, and then the eluate was collected again. These two eluates were merged together to detect the concentrations of Fe and Sb (III) by atomic absorption spectrometry. Subsequently, the used sorbents were washed with 10 ml of distilled water twice before reusing. The recycling was repeated for another two rounds, and all the results are listed in Table 2, demonstrating that the acid-resistant magnetic Fe_3_O_4_ adsorbents may still function after being reused at least three times. Moreover, the adsorbed antimony could be eluted by using 1 M HCl and 1 M H_3_PO_4_, without iron ions leached. In other words, the acid-resistant magnetic Fe_3_O_4_ adsorbents prepared by our method are economical and potentially applicable to antimony (III) removal under strong acid conditions.

**Table 2.** Sb(III) concentrations after adsorption, Sb(III) concentrations in eluate and Fe concentrations in eluate for three recycles.

Round	Sb(III) concentration after adsorption(mg l^–1^)	Sb(III) concentration in eluate (mg l^–1^)	Fe concentration in eluate
1	55.4	146	nil
2	67.3	121	nil
3	75.6	107	nil

## 4. Conclusions

In this study, a new facile strategy has been successfully developed to protect nano-sized magnetite particles from corrosion by strong acid. This strategy was based on the hydrophobic property of methyl groups originating from the hydrolysis of DMDES. Through three different sol-gel processes at 30°C, the nano-sized Fe_3_O_4_ particles were coated by three corresponding shells with a structure of Fe_3_O_4_@SiO_2_@SiO-Me@SiO-NH_2_. The middle shell was full of methyl groups that resisted acid corrosion, and abundant amino groups, which were convenient for further modification, existed in the outmost shell. To adsorb Sb(III) in the acid solution, amino-methylene-phosphonic groups were modified on the adsorbent surfaces as chelating groups. As expected, the acid-resistant magnetic Fe_3_O_4_ nanoparticles selectively removed Sb(III) from cupric sulfate solution with 2.5 M sulfuric acid, and also could be reused at least three times. These findings prove that the coated magnetite particles modified with amino-methylene-phosphonic group can be applied in the purification of copper refining electrolytes by the MSPE process. The adsorption of Sb(III) from 2.5 M sulfuric acid solution needed four hours to reach equilibrium. Our coating strategy, with the chelating groups changed, may also be suitable for the adsorption of other metal ions in acid condition. Furthermore, it is feasible to protect other types of materials from strong acid corrosion with this method.

## Disclosure statement

No potential conflict of interest was reported by the authors.
